# Psychological and behavioural responses to death anxiety in older adults with chronic illnesses: a systematic integrative review

**DOI:** 10.3389/fpsyg.2025.1684385

**Published:** 2025-12-10

**Authors:** Leovaldo Alcântara, Teodora Figueiredo, Luís Midão, Elísio Costa, Constança Paúl

**Affiliations:** 1 School of Medicine and Biomedical Sciences, University of Porto, Porto, Portugal; 2 Department of Education and Psychology, University of Aveiro, Aveiro, Portugal; 3 RISE-Health, Competence Center for Active and Healthy Aging, Faculty of Pharmacy, University of Porto, Porto, Portugal; 4 RISE-Health, Department of Behavioural Sciences, School of Medicine and Biomedical Sciences, University of Porto, Porto, Portugal

**Keywords:** mortality salience, older adults, chronic disease, treatment adherence, existential reflection

## Abstract

**Background:**

Death anxiety has been identified as a relevant emotional response in older adults with chronic illness, but its role in shaping psychological and behavioural responses remains underexplored.

**Objective:**

This integrative review aimed to investigate how death anxiety influences the emotional and behavioural responses of older adults living with chronic conditions, and to identify theoretical and clinical implications.

**Methods:**

A systematic search was conducted across PubMed, Scopus, Web of Science, and PsycINFO databases, using an integrative review framework. Nine studies met the inclusion criteria and were analysed using thematic synthesis and quality appraisal.

**Results:**

Death anxiety was associated with both negative and positive or adaptive outcomes, including emotional distress, avoidance behaviours, increased treatment adherence, and re-evaluation of personal values. Thematic analysis revealed five key domains: (1) psychological responses; (2) behavioural responses; (3) predictors and moderators; (4) spirituality as a buffer; and (5) assessment strategies.

**Conclusion:**

Death anxiety plays a central and context-dependent role in shaping health-related behaviours in later life. Its effects are moderated by factors such as spirituality, social support, disease burden, and emotional resilience. These findings support the development of integrative clinical models that combine existential and behavioural frameworks to improve adherence and well-being in older populations.

## Introduction

The global number of individuals aged ≥65 is expected to double by 2050 to encompass 2.1 billion, and population ageing is therefore one of the key phenomena in the 21st century (Sun and Li, 2023; Grinin et al., 2024). Nevertheless, chronic non-communicable diseases are common in this population and create major management challenges (Sun and Li, 2023). Chronic diseases represent the leading causes of death worldwide, as reported by the World Health Organisation (WHO) (WHO, 2003; Wilder et al., 2021). However, the treatment adherence among older adults with chronic diseases remains particularly unsatisfactory and only half of them adheres to prescribed indications, according to the WHO (WHO, 2003; Baryakova et al., 2023; Gautam et al., 2021). Enhancing compliance with medical treatment is one of the major public health concerns and problems worldwide (Gautam et al., 2021; Bartlett Ellis et al., 2023). Non-adherence suggests additional costs in public healthcare policies drives the probability of another comorbidity, decreases the resources and quality of life, and functionality of patients (Prakash et al., 2021; Mendoza Reyes, 2021). Adherence is especially critical in chronic diseases due to the need for prolonged treatments, lifestyle and routine changes, polypharmacy, and the cumulative functional impact resulting from the disease, as well as, in many cases, the adverse effects of treatments (Religioni et al., 2025).

Ageing should be understood as a natural and multifaceted process resulting from the interaction of biological, psychological and social factors. When associated with chronic diseases, this process is often accompanied by challenges related to health, functionality and autonomy. In this context, the concept of active ageing — understood as the optimisation of opportunities for health, participation, security and lifelong learning — is central to promoting well-being in old age (Menassa et al., 2023). Quality of life in old age is intrinsically linked to maintaining independence, cultivating social ties and engaging in meaningful activities, as well as the ability to adapt to the physical and emotional changes that are characteristic of this stage of life (Goodarzi et al., 2024). Integrating existential dimensions, such as anxiety about death, into the analysis of these processes can contribute to the improvement of clinical and psychosocial models aimed at therapeutic adherence and the promotion of healthy ageing.

The definition of adherence has changed over the years. Adherence is defined in this study as conscious compliance with advice from healthcare professionals, which may involve altered routines, habits, or medication use (Bartlett Ellis et al., 2023; Foley et al., 2021; Nunes, 2020; Cruz, 2017).

Some factors have been described as important with regard to adherence to treatments and which may represent barriers or facilitators. These factors are patient-related, disease-related, treatment-related, healthcare-related, professional-related as well as cultural and sociodemographic attributes (Foley et al., 2021; Kvarnström et al., 2021). Although there has been an increasing trend in the study and investment in adherence, the rates have remained largely unchanged for more than two decades (Kardas et al., 2024). This lack of progress may indicate that current research does not provide a complete explanation of all determinants of adherence, particularly in terms of subjective determinants (Cemin et al., 2024). Chronic illness in old age can trigger negative emotions and existential reflection on the end of life, and can lead to symptoms of anxiety (Emmerich, 2024). Death anxiety, mortality salience, mortality awareness, attitude toward death, and death-related thoughts are the terms that have been commonly used to refer to the state in which people focus on their own death (Bluck et al., 2022; Çayırcı et al., 2024). However, the literature on how mortality awareness specifically influences adherence remains scattered and inconclusive.

This gap is particularly evident in three areas. First, most existing studies on death anxiety and treatment adherence focus on younger or middle-aged adults, with limited attention to older populations living with chronic illness (Rich et al., 2016). Second, psychological and existential factors are rarely integrated into adherence models, which tend to prioritise clinical and behavioural determinants (Rapoff et al., 2023). Third, key constructs such as death anxiety and mortality salience are often used interchangeably, despite representing distinct psychological phenomena with different behavioural implications (Routledge and Juhl, 2010). These limitations underscore the need for a more integrative and age-sensitive approach to understanding treatment adherence.

Although isolated studies have explored this relationship, recent meta-analyses reinforce the clinical and theoretical relevance of death anxiety in chronic disease contexts. For example, a systematic review identified robust associations between death anxiety and psychological distress in adults with chronic illnesses, including depression, generalised anxiety, and poor treatment adherence (Dumitru et al., 2025). Another meta-analysis demonstrated the effectiveness of psychosocial interventions, such as logotherapy and cognitive-behavioural therapy, in reducing death anxiety in patients with chronic diseases (Gulbahar Eren et al., 2025). These findings reinforce the need to integrate existential dimensions into therapeutic adherence models, especially in ageing populations.

In addition to clinical and behavioural factors, it is necessary to consider subjective and existential dimensions that influence adherence, such as death anxiety.

Initially, it is necessary to clarify three interrelated but distinct ideas. First, there is finitude, which refers to the reality of mortality, a condition inherent to all living beings, and the way we experience time as something limited (Király and Köves, 2023; Carstensen et al., 1999). Next, there is the awareness of mortality, which is the conscious or semi-conscious understanding that our own death is not just a possibility but an inevitable reality (Doka, 2021; Huang et al., 2021). Finally, there is death anxiety, the emotional reaction—ranging from fear to total denial—that accompanies this awareness (Çayırcı et al., 2024; Karaman et al., 2025). Although these terms are often used as if they mean the same thing, they actually represent different layers of our existential experience and can have distinct and varied impacts on our behaviour.

These distinctions are not merely theoretical; they have practical implications in clinical contexts. Finitude, as a broader concept, refers to the understanding of life as temporarily limited. For example, an individual may recognise that ageing and the accumulation of health problems increase the likelihood of death — not just as a possibility, but as an approaching reality — evoking awareness of their own mortality (Carstensen et al., 2003). Anxiety about death, in turn, arises as an affective response to this awareness, often manifesting as fear of suffering, loss of autonomy, or existential angst (Dursun et al., 2022). In the ageing process, particularly in old age, the onset of a chronic illness can trigger a variety of emotional reactions, depending on how the individual processes the notion that their time is finite and that the illness may shorten or prolong the inevitable encounter with death (Awang et al., 2018). These affective responses may include anxiety, distress, or avoidance. Avoidance, in particular, can manifest as denial or disengagement from treatment, thus compromising adherence (Baker, 2009). On the other hand, individuals who accept their condition may feel motivated to engage in treatment as a means of preserving life and prolonging a meaningful existence (Blomstrom et al., 2020).

To understand these reactions, it is essential to resort to theoretical models such as Terror Management Theory (TMT), which offers an explanatory lens on how we deal with the awareness of finitude.

One of the main frameworks for understanding how we respond to the awareness of our mortality is Terror Management Theory (Pyszczynski et al., 2021; Shakil et al., 2022). This theory suggests that understanding death triggers existential anxiety, which individuals cope with by resorting to cultural beliefs and maintaining self-esteem (Chen et al., 2025). When confronted with reminders of mortality, such as when facing illness or as they age, people may cling to healthy habits to regain a sense of control and meaning, or, on the other hand, they may resort to avoidance, denial, or ignorance of health advice as a way to defend themselves (Gyimes and Valentini, 2023). Although TMT is commonly used, other theories also provide insights into how people think about death and mortality. For example, Frankl (1985) argued that we have the ability to find meaning even in suffering. Meanwhile, existential psychology, as well as the Meaning Management Theory proposed by Paul T. P. Wong in 2008, explore how confronting finitude can lead to personal growth, acceptance, or suffering, depending on an individual’s psychological resources and worldview (Wong, 2007). These complementary frameworks help to broaden our understanding of death anxiety, especially in the context of chronic illness and ageing.

Considering these theoretical approaches, this integrative review aims to investigate how death anxiety influences the psychological and behavioural responses of older adults with chronic diseases, with particular attention to the implications for treatment adherence and existential well-being. To date, no integrative review has simultaneously addressed the concepts of death anxiety, therapeutic adherence, and ageing in populations with chronic diseases, which reinforces the originality and relevance of this study. By thematically organising the available empirical evidence, this review aims to contribute to the development of clinical models that are more sensitive to the existential dimensions of chronic disease management, with the potential to optimise both therapeutic adherence and psychological well-being in ageing contexts

## Methods

This review is integrative, according to the method of Whittemore and Knafl (2005), and was chosen because it allows for the inclusion of studies with different methodological approaches (quantitative, qualitative, and theoretical), which is essential for understanding a multifaceted phenomenon such as anxiety about death. This study adheres to the PRISMA checklist (Page et al., 2022) to ensure methodological transparency. The phases included the following: (I) Identification of the topic and formulation of the research question; (II) Establishment of inclusion and exclusion criteria; (III) Literature search; (IV) Critical evaluation of selected studies; (V) Interpretation and synthesis of results; (VI) Presentation of the integrative review (Whittemore and Knafl, 2005; Page et al., 2022). This review was not registered on the PROSPERO platform, as it is an integrative review with a conceptual focus, which includes theoretical, methodological, and empirical studies of different natures. PROSPERO is mainly focused on systematic reviews of clinical studies focusing on the effectiveness of interventions, which does not correspond to the scope of this investigation.

### Research question

What is the influence of death anxiety on psychological and behavioural responses to chronic illness in older adults?

### Search strategy

The literature search was performed in the databases PubMed, Scopus, Web of Science and PsycINFO on 05/02/2025, and included studies published up to that date. The original search string comprised controlled descriptors (MeSH, DeCS) as well as free terms combined by Boolean operators for each database (The complete search strategies are presented in Table 1): (“Death Attitudes”[Title/Abstract] OR “Mortality Salience”[Title/Abstract] OR “Death Anxiety”[Title/Abstract] OR “Death Awareness”[Title/Abstract] OR Finitude[Title/Abstract]) AND (“Chronic Illness”[Title/Abstract] OR “Chronic Disease”[Title/Abstract] OR “Chronic Condition”[Title/Abstract]) AND (elder*[Title/Abstract] OR “older adults”[Title/Abstract] OR “aging”[Title/Abstract]). The search period was not restricted and only studies published in English, Portuguese or Spanish were considered, as these are languages mastered by the authors and relevant to the research context. Only peer-reviewed articles were included; grey literature was excluded to ensure greater methodological rigour. In addition to the electronic search, manual verification of the references of the included studies was performed. The search was updated before the final analysis, and full details of the strings used are available in Supplementary Table 1.

**Table 1:** Data extracted from the articles analysed in this review.

Reference	Study type	Objective	Chronic disease	Participants	Concept related to finitude	Variables analysed	Theoretical perspective	Outcomes	Limitations of study
Bharti and Bharti (2024) India	Cross-sectional correlational survey	To explore the prevalence rate of death anxiety (DA) among older adults having chronic illness. To examine the impact of death anxiety on the psychological well-being (PWB) and successful ageing(SA) of older adults with chronic illness.	Diabetes; Hypertension; Asthma; Coronary heart disease and Arthritis	The total 79 participants comprised 43 men and 36 women, age ranged between 60 to 91 years (Mean = 69.29 years, SD = ±6.90).	Death anxiety	VDs: Psychological Well-Being (PWB); Successful ageing(SA); Demographic Factors; VI: Death anxiety;	Interdisciplinary approach	Death anxiety has a strong detrimental effect on psychological well-being and successful ageing in older adults, particularly diminishing positive emotions, relationships, and life engagement while exacerbating negative emotional states.	The sample size and data-collection method (self-reporting)
Daaleman and Dobbs (2010) USA	A cross-sectional quantitative study	To build on this work by further examining how the theoretical factors of spirituality and religiosity are associated with two death attitudes, fear of death and death acceptance, in chronically ill older adults.	Cardiovascular diseases (e.g., heart disease, heart failure, hypertension); Cerebrovascular accident (CVA); Chronic lung disease (e.g., COPD, asthma); Chronic liver disease; Chronic kidney disease	257 participants, Age (years), M (SD) 72.1 (10.1), Female 164, Male 93	Death Attitudes	VD: Fear of Death; Approach Acceptance of Death; VIs: Religiosity and Spirituality; Physical and mental health; Social support; Demographic factors	The study integrates existential psychology, theories of death and concepts of religiosity and spirituality.	Fear of death is linked to psychological distress, better physical health (paradoxically), and lack of social/spiritual support. Acceptance of death is driven by religious conviction and age, not general spirituality.	Sample characteristics and lack of specificity in one self-reported religiosity variable.
Chopik (2017) USA	Longitudinal and cross-sectional studies	Main Objective: To understand how thoughts and anxiety related to death vary throughout life and why they tend to decrease with age. econdary Objective: To investigate the role of social support and physical health in reducing death-related anxiety over time.	Hypertension; Diabetes; Cancer; Lung disease; Coronary heart disease, including: Heart attacks; Angina; Congestive heart failure; Emotional, nervous or psychiatric problems; Arthritis or rheumatism; Cerebral vascular accident (CVA).	Study 1: Participants were 2,363 adults ranging in age from 18 to 88 years (M = 36.17, SD = 13.53; 70.2% were women); Study 2: Participants were 9,815 older adults, ranging in age from 50 to 96 years (M = 67.41, SD = 9.08; 59.1% were women)	Death-related thoughts and Death Anxiety	VDs: Thoughts about death; Anxiety related to death; VIs: Age; Social support; VCs: Self-reported health, Chronic diseases, Gender	Terror Management Theory (TMT) and Socioemotional Selectivity Theory (SST)	Ageing reduces death anxiety when health is taken into account, but declining health exacerbates it. Social connections - especially intimate relationships - act as a protective buffer over time. Gender plays no measurable role.	In the longitudinal study, anxiety about death was measured by a single item. Another limitation is the way in which the index of chronic illnesses was created.
Rezaei Aderyani et al. (2021) Iran	Cross-sectional study	To determine the relationship between spirituality and death anxiety in the older adults with chronic disease.	Heart disease; Hypertension; Diabetes; Kidney failure; Asthma	150 patients over the age of 60. The mean age of the subjects was 63.26 years (SD = 7.62). Most of them were married (73.6%) and 50.7% were male	Death anxiety	VD: Death anxiety; VIs: Spirituality (meaning, peace, faith), anxiety, depression, gender, age, marital status, educational level, economic status, chronic diseases, duration of illness, BMI, smoking history.	A multidisciplinary theoretical framework, integrating concepts from psychology, gerontology, spirituality and health.	Moderate levels of death anxiety and relatively high spirituality scores, with faith emerging as the strongest spiritual component and peace as the weakest. Gender differences were notable, with women showing significantly higher death anxiety than men. More spiritual individuals tended to experience less death-related distress. This inverse relationship held consistently across all the spirituality components measured. Two significant predictors of reduced death anxiety: stronger faith and lower levels of general anxiety.	Self-reported data collection, limited to only one city in Iran with religious and Muslim people and care should be taken in generalising the results.
Doğan and Hacıköylü (2025) Turkey	It is a cross-sectional and correlational study	To determine the attitudes towards ageing and death anxiety levels and the relationship between them in individuals aged 65 and over with chronic diseases.	Cardiovascular diseases; Hypertension; Diabetes; Chronic respiratory diseases; Cancer; Other conditions such as: Kidney failure; Prostate problems and Osteoporosis.	A total of 169 individuals aged 65 years and over with chronic diseases participated in the study. It was determined that 72.2% of the individuals participating in the study were in the 64–74 age group, 63.9% were female, 55.6% were primary school graduates, 78.1% were married.	Death anxiety	VDs: Death Anxiety and Attitudes toward death, VIs: Sociodemographic factors; Clinical factors; Psychosocial factors	A multidisciplinary theoretical framework, integrating concepts from gerontology, the psychology of ageing, theories of death and spirituality, and public health.	Women reported significantly higher death anxiety than men. Poor self-assessment of health status was associated with much higher anxiety. People with higher expenses than income have higher anxiety. ‘Feeling unhappy/desperate’ predicted higher anxiety. Negative attitudes towards ageing: Older adults in villages had more negative opinions than their urban peers. Lower income than expenses aggravated attitudes. 92.3% of sexually inactive individuals had more negative attitudes.	The results of the study can only be generalised to individuals over the age of 65 with chronic diseases. The questionnaires and scales used in the study were high in terms of the number of items considering the older individuals.
Guo et al. (2024) China	Longitudinal Survey	To investigate the relationship between the number of chronic diseases and death anxiety in the older adult (≥65 years) in rural China.	Hypertension; Diabetes; Heart disease (e.g., heart failure, coronary heart disease); Cerebrovascular accident (CVA); Cataracts or glaucoma; Cancer or malignant tumours; Bronchitis or other chronic respiratory diseases; Arthritis or rheumatism; Stomach problems (e.g., gastric ulcer); Osteoarthritis or osteoporosis; Liver, gallbladder or bladder disease; Dementia.	The mean age of observations was 71.08 years old, and the mean score for death anxiety was 3.27. There were slightly more men than women (51.87%), and most of the participants had no formal education (60.39%) and were married (70.16%), received caregiving from others (60.41%), no longer worked (52.19%), and had no religious beliefs (83.43%).	Death anxiety	VD: Death anxiety; VIs: Number of chronic conditions; Age	Terror Management Theory - TMT; Theory of Resilience in Ageing	Inverted U-shaped curve: Peak anxiety: older adult with 2–3 chronic illnesses had the highest levels of anxiety (*β* = 0.28, *p* < 0.001). Reduction after 4 + illnesses: Anxiety decreased significantly (*β* = −0.03, *p* < 0.001), suggesting psychological adaptation (resilience). Moderating effect of age Younger older adult (65–74 years): Showed greater sensitivity to increased illness (anxiety rose rapidly). Peak anxiety occurred with fewer illnesses (2–3). Very older adult (≥85 years): Anxiety less affected by the number of illnesses (flatter curve). Significant interaction: No. of illnesses × age: *β* = −0.02 (*p* < 0.05). (No. of illnesses)^2^ × age: β = 0.002 (*p* < 0.05).	Self-reporting scales were used and a rural sample, so results may not be generalisable to urban areas.
Özteke Kozan and Kesici (2023). Turkey	Phenomenological qualitative research	To examine the psychological effects of Covid-19 on the older adult with chronic illnesses living in Turkey with a phenomenological research orientation.	Chronic asthma; Hypertension; Chronic asthma-tachycardia; Diabetes; Cardiovascular disease; Renal impairment; Cancer; Cardiac dysrhythmia	A total of 18 older people aged 65 and over, aged between 65 and 84. There were 10 females and 8 males.	Death anxiety	Theme 1. The meaning of death before Covid-19; Theme 2. Meaning of death during Covid-19; Theme 3. Awareness about life; Theme 4. Anxiety toward family members during Covid-19; Theme 5. Effects of Covid-19 on daily life; Theme 6. Future anxiety after Covid-19; Theme 7. Coping strategies for death anxiety	Terror Management Theory (TMT), Attachment theory, and existential theory.	The findings suggest that for this population, death anxiety during COVID-19 was: (1) more relational than self-focused, (2) tied to specific disease characteristics, and (3) mediated by both spiritual frameworks and practical behavioural strategies. Notably, participants employed multiple adaptive strategies to manage death anxiety, like increased family time or productivity. Spirituality served as a key coping resource, alongside concrete activities like new hobbies.	The study is limited to older people with chronic illnesses.
Ji et al. (2024) China	Quantitative cross-sectional study	To investigate of the mediating relationship between death anxiety, self-esteem and quality of life.	Vascular diseases; Endocrine/metabolic diseases (e.g., diabetes); Breast cancer; Liver diseases; Heart disease	Out of 294 patients with chronic diseases. 108 (36.74%) were female and 186 (63.26%) were male; the age range of the participants was 45–96 years with a mean age of 64.06 ± 10.23 years	Death anxiety	VD: Quality of life; VI: Self-esteem; VM: Death anxiety	Terror Management Theory (TMT); Attachment theory; Biopsychosocial Model	Higher self-esteem predicts better-reported QoL. Individuals with higher self-esteem experience lower death anxiety. More significant death anxiety correlates with poorer QoL. Death anxiety mediates 18.4% of the total effect of self-esteem on QoL. Self-esteem retains a strong independent influence on QoL (81.6% of total impact). The link between self-esteem and QoL is partly explained by reduced death anxiety.	To lessen interference with the findings, tighter control over other variables should be used in future studies. Second, this study could only represent one region because it used sample data from shejiang Province.
Comertoglu et al. (2024) Turkey	A cross-sectional study	To examine the prevalence of social frailty in Turkey and its relationship with quality of life and the mental health of older adults. It also aimed to determine the factors that indicate social frailty.	Cardiac; Vascular; Haematopoietic; Respiratory; Otorhinolaryngological/Ophthalmological; Gastrointestinal; Hepatic; Genitourinary; Musculoskeletal; Neurological; Endocrine/Metabolic; Psychiatric; Breast.	This study included 136 participants in total. The median (min–max) age was 72 (65.3–90.3) years. A total of 38.2% of the participants were aged 75 and older	Death anxiety	VD: Social Frailty VIs: Psychological Factors: (Anxiety, Depression, Death anxiety, Loneliness) Clinical Factors: (Burden of Chronic Illness; Physical Frailty) Sociodemographic factors: (Marital status, Household arrangement, Level of income and schooling)	It is anchored in interdisciplinary concepts of gerontology, public health, with an emphasis on the Social Frailty Theory.	Death anxiety clusters with social frailty, loneliness, and psychological distress, exacerbating quality-of-life declines. Socially frail participants showed significantly higher death anxiety compared to robust peers. Co-occurred with depression and moderate/severe anxiety. Frail individuals with death anxiety had lower QoL.	The study may not represent the general geriatric population in Turkey, only patients who were admitted to a university hospital’s geriatric outpatient clinic were included.

### Inclusion and exclusion criteria

The PCC approach (Pollock et al., 2023) was adopted to guide the inclusion and exclusion criteria, P (Population): Older people aged 60 years and over with long-term chronic diseases needing long-term treatment; C (Concept): Awareness about finitude, thinking of death, awareness of death, death anxiety or existential considerations in the context of chronic disease management; C (Context): Psychological and behavioural factors associated with the awareness of finitude, thinking of death, awareness of death, death anxiety or existential considerations in chronic health conditions. In the screening of titles, abstracts and full texts, those that met one or more of the following exclusion criteria were excluded: Studies that did not target older adults; Studies that did not provide information about awareness of finitude, mortality salience, death anxiety or existential reflections; Studies that did not target chronic conditions, chronic illness and chronic diseases or non-communicable diseases; Studies that focused exclusively on physical or biological aspects of chronic condition without psychological or existential levels being considered in chronic condition; Studies that did not report findings about psychological and behaviour determinants related to chronic condition; Studies published neither in English, Portuguese nor Spanish.

The age criterion of ≥60 years was adopted because this is the age group in which chronic diseases become most prevalent (Ansah and Chiu, 2022), in addition to corresponding to the definition of older adult in developing countries. Studies with mixed samples were included only when they presented specific data for participants aged 60 years or older. Although the main focus was on studies that used the term “anxiety about death,” studies that addressed related concepts, such as “awareness of finitude, salience of mortality, thoughts related to death, or existential reflections,” were also considered, provided they were directly linked to psychological distress in the face of death in the context of chronic diseases. These concepts are interrelated and related to the theme of finitude (Dursun et al., 2022).

### Data extraction and synthesis

**Figure 1:**
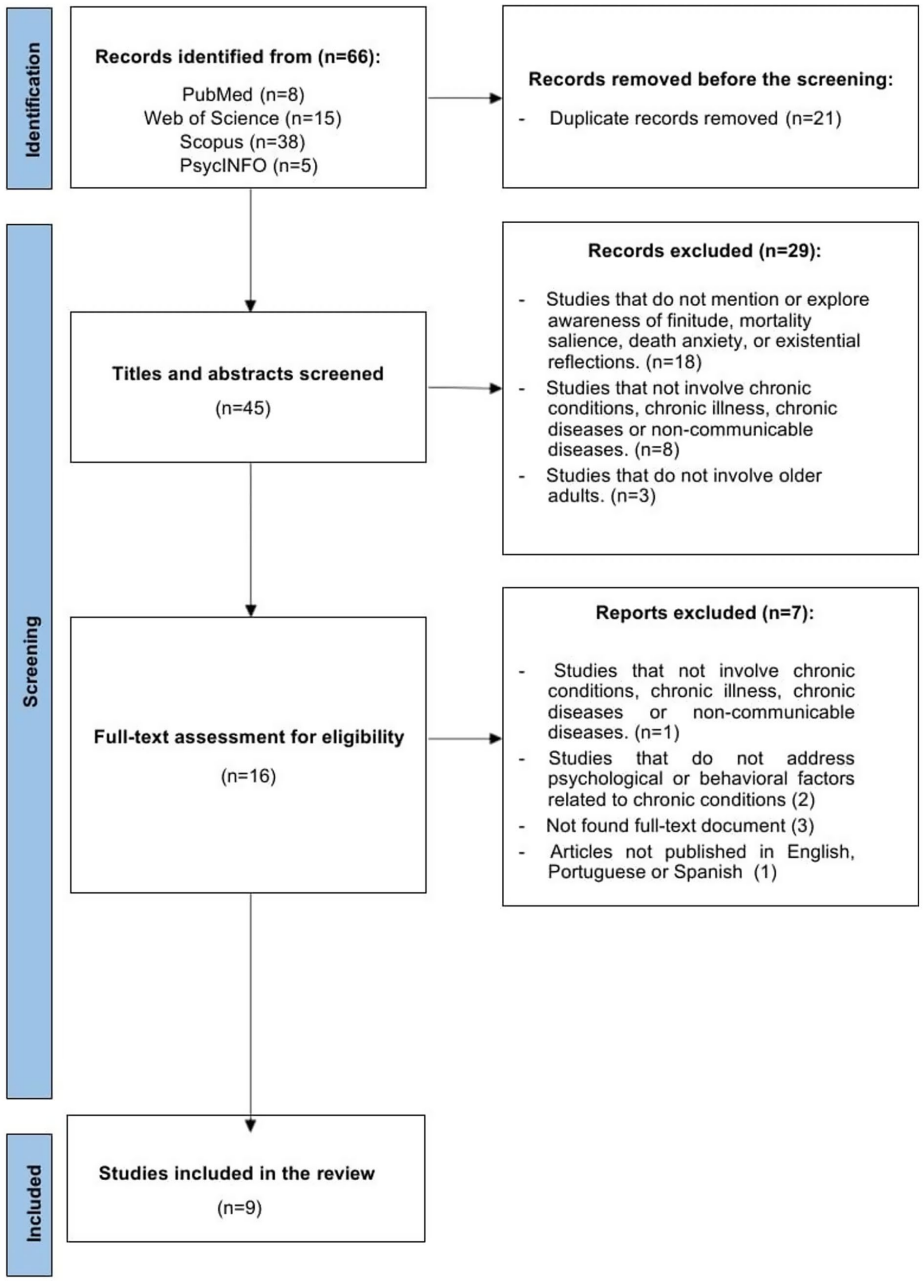
Articles’ screening process based on the PRISMA flow diagram.

Following duplication removal, two reviewers, L. A. and T. F., independently assessed the articles, first by screening title-and-abstract and finally full-text screening. The agreement between the two independent reviewers was assessed using Cohen’s Kappa coefficient. Decisions to include or exclude articles were coded as binary (include = 1, exclude = −1). Only articles evaluated by both reviewers were considered in the analysis. The Kappa value was calculated for both screening phases: in the title-and-abstract phase, the resulting value was 0.25, indicating only fair agreement according to Landis and Koch’s classification (Landis and Koch, 1977). In the full-text screening phase, the Kappa value was 0.61, indicating substantial agreement between reviewers in this specific phase. Although the initial agreement was low, further calibration meetings and explicit decision rules increased consistency in later phases. Furthermore, all discrepancies were resolved through discussion and consensus between the two reviewers and an additional reviewer, E. C., ensuring consistency in the final selection. The selection process is detailed in the PRISMA flowchart diagram (Figure 1).

Data extraction for the studies in this review occurred using a standardised form specifically designed for this review available in Appendix 1. The form contained the following domains: title, author, country, year, study design, aims of the study, chronic disease, characteristics of participants (age, gender and sample size), concepts of finitude, variables explored (independent and dependent), theoretical frame, key findings, and limitations of the study. This review utilized Rayyan (Ouzzani et al., 2016) to screen and organized the selected studies using Excel and EndNote (Analytics, 2023). In order to maintain accuracy, the synthesis processes were guided by those suggested for integrative reviews (Whittemore and Knafl, 2005). A narrative approach was employed for data synthesis, justified by the heterogeneity of the studies included, both in terms of methodological design (qualitative, quantitative, and theoretical) and contexts and populations. After extraction, the data were organised into conceptual categories that corresponded to the theoretical focus of the review. These categories were not predefined, but rather emerged inductively with data familiarisation and refined through an iterative process. The studies were organised based on their main theoretical orientation — existentialist, behavioural, or integrative — as well as the psychological and behavioural outcomes they addressed, such as coping strategies, adherence behaviours, and emotional responses. We also considered how each study viewed mortality awareness or the concept of finitude. This form of categorisation enabled the use of a thematic synthesis approach, facilitating the identification of patterns and differences between studies. We carried out the synthesis narratively, following a theory-building logic that allowed us to weave empirical insights into a cohesive conceptual model (Popay et al., 2005).

### Quality appraisal

The aim of the quality assessment was not necessarily to reject studies, but rather to characterise the quality of the studies included in this review. To assess the quality of the evidence of the studies, the Critical Appraisal Tools of the Joanna Briggs Institute were employed (Joanna Briggs Institute, 2020). With the instruments the quality of the studies (Bharti and Bharti, 2024; Daaleman and Dobbs, 2010; Chopik, 2017; Rezaei Aderyani et al., 2021; Doğan and Hacıköylü, 2025; Guo et al., 2024; Özteke Kozan and Kesici, 2023; Ji et al., 2024; Comertoglu et al., 2024) were independently evaluated by the two authors (L. A., T. F.) and discrepancies were discussed. Assessment of study quality with specific checklists according to each individual study type showed that most studies met all criteria and could be considered to have good quality evidence (see Supplementary Table 2). There was one study in which the exposure assessment was not valid or reliable, the measurement of results was not done with objective and standardised criteria, confounding factors were not considered and techniques to minimise confounding were not mentioned (Bharti and Bharti, 2024). Moreover, in the longitudinal study (Guo et al., 2024) did not complete the follow-up process and specific reasons were not reported, no clear strategy was employed to handle incomplete follow up but replacement of participants in later waves was referred. In the cross-sectional and longitudinal study, used a single-item measure to assess death anxiety, culminating in limiting the validity of the result (Chopik, 2017). Most studies were of good methodological quality, although some had limitations, such as the use of unvalidated measures, lack of control for confounding factors, or unjustified loss to follow-up. A formal scoring system was not used, but rather a narrative assessment based on the criteria of the instruments. Given the conceptual objective of the review, no studies were excluded due to methodological limitations, but these were explicitly considered in the interpretation of the results.

### Ethical considerations

No ethical approval was required as this study is based on previously published literature. All data extracted are from publicly available sources. Protocols, search strategies, and extraction forms are available upon request.

## Results

The database search during the materials selection phase yielded 66 articles, 11 of which were duplicates and they were removed. After reading the titles and abstracts, 29 were excluded, and the full text of 16 studies was read. Nine studies were classified as eligible and included in this review.

### Study characteristics

The last 5 years were the most productive period, with most publications on the subject emerging (*n* = 7) in this period. The included studies were conducted in five countries: Turkey (*n* = 3), China (*n* = 2), United States of America (*n* = 2), Iran (*n* = 1), and India (*n* = 1).

Regarding the study design, most were quantitative cross-sectional studies (n = 6), followed by one quantitative longitudinal study, one mixed-method study, and one qualitative study employing a phenomenological approach.

Study sample sizes ranged from *n* = 18 (Özteke Kozan and Kesici, 2023) to *n* = 9,815 (Chopik, 2017), all of whom were older adults. The mean age of participants across studies varied between 60 and 96 years. The overall average mean age was 70 years, with the highest mean age (72.10) reported in study (Daaleman and Dobbs, 2010) and the lowest (64.06) in study (Ji et al., 2024).

Of the nine included studies, three (Bharti and Bharti, 2024; Guo et al., 2024; Ji et al., 2024) reported a higher proportion of male, while the remaining six included a predominance of female participants. Eight of the nine studies included participants with multiple chronic conditions, whereas one study (Chopik, 2017) focused exclusively on individuals with a single morbidity (see Table 1 for full data extraction).

### Death anxiety and chronic illness

The reviewed studies indicated that experiencing deterioration in physical health highlights one’s likelihood to face decline and death that may lead to the mobilisation of self-care behaviours or to resistance and denial (Bharti and Bharti, 2024; Daaleman and Dobbs, 2010; Chopik, 2017; Rezaei Aderyani et al., 2021; Doğan and Hacıköylü, 2025; Guo et al., 2024; Özteke Kozan and Kesici, 2023; Ji et al., 2024; Comertoglu et al., 2024). The studies reviewed indicated that diseases such as cardiovascular disease, cancer and diabetes have been found to be associated with higher levels of death anxiety (Bharti and Bharti, 2024; Doğan and Hacıköylü, 2025). Anxiety is highest in patients with 2–3 chronic diseases, and decreases with 4 or more diseases (Guo et al., 2024). The onset of chronic illness may serve as a reminder of one’s inevitable mortality, heightening awareness of finitude and intensifying death-related anxiety (Guo et al., 2024). In addition, lower self-reported health had a positive relationship with death anxiety in one study (Doğan and Hacıköylü, 2025). Conversely, in another study, people reporting increasingly better health had more death-related concerns, which indicates that this is also a fear of losing one’s independence (Daaleman and Dobbs, 2010).

Social fragility, indicated by low levels of education, financial difficulties and comorbidities, was identified as a context-specific factor affecting how awareness of one’s own mortality is faced and was also associated with higher death anxiety and more difficulty in managing the chronic health condition (Daaleman and Dobbs, 2010).

It is important to distinguish between awareness of mortality—the cognitive recognition of a person’s finite existence—and death anxiety, the affective response that this awareness can provoke. Although several studies implicitly treat these concepts as overlapping, they refer to different psychological phenomena (Maxfield et al., 2007; Neimeyer, 2015). Awareness of mortality can exist without death anxiety, and vice versa.

Furthermore, although illness may increase the salience of mortality, awareness of death is not unique to illness. According to the Theory of Socioemotional Selectivity (Carstensen, 2021), ageing is often accompanied by a shift on time perspective. This shift often leads us to prioritize emotionally significant goals and reassess what truly matters in life (Carstensen et al., 1999). This change in perceived time remaining is influenced not only by chronological ageing but also by significant health events throughout the lifespan (Carstensen et al., 1999; Carstensen, 2021). Over time, such changes can increase an individual’s awareness of mortality, even in the absence of illness (Carstensen et al., 2003).

These intensified existential concerns, especially when unresolved, can give rise to a range of psychological and behavioural reactions. These are explored in the following sections.

### Psychological responses to death anxiety

All nine studies included in this section focused on mortality-related constructs such as death anxiety, attitudes toward death, and death-related thoughts. The theoretical frameworks underpinning in the analysed studies reflected two broad explanations of the association between mortality awareness and health-related behaviours: an existential explanation, based on the search for meaning, spirituality, and acceptance present in six studies (Daaleman and Dobbs, 2010; Chopik, 2017; Rezaei Aderyani et al., 2021; Doğan and Hacıköylü, 2025; Özteke Kozan and Kesici, 2023; Ji et al., 2024). In contrast, a behavioral explanation, focused on the prediction and explanation of health-related behaviours, including treatment adherence, was identified in three studies (Bharti and Bharti, 2024; Guo et al., 2024; Comertoglu et al., 2024).

In the study (Bharti and Bharti, 2024), the death anxiety was found to have a strong negative impact on psychological well-being and successful ageing in the older adult, especially reducing the elements of positive emotions, relationships and life engagement, and increasing negative emotional conditions. Similarly, study (Daaleman and Dobbs, 2010) reported high levels of death anxiety were reported in persons who have lower levels of intrinsic religiosity and high levels of depression. A mixed-methods study (Chopik, 2017) analysing coping strategies revealed that the participants encountered high emotional burden faced with the thought of their death, including indirect (e.g., get out of talking about it) and direct manifestations (e.g., refusal to plan for the future).

In the study (Doğan and Hacıköylü, 2025), experiencing unhappiness/despair emerged as a significant predictor of death anxiety and negative attitudes toward ageing. In the study (Guo et al., 2024), was found that the worse the health condition (for example, multimorbid entails), the higher the fear of death, the inability to regulate emotions, the worse ability to follow the treatments correctly. In the study (Ji et al., 2024), the extent of death anxiety was negatively associated with the poorest self-esteem and well-being of those with cancer. Finally, the study (Comertoglu et al., 2024) found that death anxiety was accompanied by social fragility, loneliness and psychological distress, even worsening the decrease in quality of life and exacerbating coexisting depression and emotional vulnerability.

### Behavioural responses to death anxiety

The integrated review of the included studies shows that awareness of mortality evokes distinct behavioural responses among older adults with chronic diseases. These reactions are shaped by psychological, spiritual, and contextual resources and can function as either facilitators or inhibitors of health-related behaviours.

In study (Ji et al., 2024), individuals with lower levels of death anxiety were more likely to adhere to medical treatment, medical appointments, and other self-care behaviours. Conversely, studies (Bharti and Bharti, 2024; Rezaei Aderyani et al., 2021) found that in situations with a high degree of threat, or when there is insufficient existential elaboration, death anxiety can provoke avoidant behaviors, for example, refusing invasive treatments, not attending hospital appointments, not recognizing the severity of the condition. This was more frequent among participants with low social/spiritual support and high symptoms of anxiety, similar to depression.

In studies (Chopik, 2017; Rezaei Aderyani et al., 2021; Özteke Kozan and Kesici, 2023) participants who experienced mortality confrontations described reassessing their life priorities, including placing greater value on their relationships or spirituality or on quality of life over quantity. For example, in some cases, participants chose to refuse active treatment in order to maintain dignity or independence. The study (Daaleman and Dobbs, 2010), further demonstrated that individuals with higher self-efficacy and social support were more likely to adopt health-promoting behaviours.

### Predictors and mediators of death anxiety

Across the reviewed studies, several factors were consistently associated with higher levels of death anxiety. These included social frailty, isolation from social life, mental distress, the deterioration of quality of life, high level of general anxiety and depression, low physical functioning and poor self-rated health, lower education, the number of diseases, female gender, younger age, living in rural areas, and lower level of spiritual involvement or religiosity (Bharti and Bharti, 2024; Daaleman and Dobbs, 2010; Rezaei Aderyani et al., 2021; Guo et al., 2024; Ji et al., 2024; Comertoglu et al., 2024).

Several variables were also identified as mediators or moderators of the relationship between death anxiety and its emotional or behavioural outcomes. These included a strong sense of meaning in life, engagement in spiritual practices, perceived control and coping ability, emotional and instrumental social support, higher levels of education, and a positive sense of self-worth (Bharti and Bharti, 2024; Daaleman and Dobbs, 2010; Rezaei Aderyani et al., 2021; Ji et al., 2024). These findings indicate that both intrapersonal and situational resources can help mitigate the psychological and behavioural effects of mortality awareness.

### Spirituality and death anxiety

In addressing death anxiety, spirituality has become an important coping resource and source of strength, especially in coping ability of older adults with chronic disease. The study (Ji et al., 2024) found that the higher the spirituality of the faith-based sample, the lower the death anxiety and the greater the well-being. Similarly, the study (Daaleman and Dobbs, 2010) found that individuals’ meaning of life and death meaning were associated with great treatment adherence, suggesting a moderating role of spiritual beliefs.

Study (Özteke Kozan and Kesici, 2023) shed light on the protective role of spirituality, revealing that engaging in spiritual practices can serve as a psychological shield—promoting inner peace and boosting resilience when facing illness. In the study (Rezaei Aderyani et al., 2021), spirituality was examined in the context of death anxiety, and religiousness became a significant approach for dealing with anxiety as well as the most predictive domain of spirituality. Additionally, stronger faith was related to higher levels of acceptance of finitude and lower general anxiety (Rezaei Aderyani et al., 2021).

### Assessment tools for death anxiety

The nine reviewed studies measured death anxiety with a variety of instruments. The most commonly used was the Templer Death Anxiety Scale (DAS), used in four studies (Bharti and Bharti, 2024; Rezaei Aderyani et al., 2021; Guo et al., 2024; Comertoglu et al., 2024). Modified versions of the DAS were also employed: the Turkish Death Anxiety Scale (TDAS) in study (Doğan and Hacıköylü, 2025), and the Chinese version (CT-DAS) in study (Ji et al., 2024).

The study (Daaleman and Dobbs, 2010) used two instruments to assess multidimensional nature of death-related attitudes: the Death Attitude Profile-Revised (DAP-R) and the Collett-Lester Fear of Death Scale. The study (Chopik, 2017), measured death anxiety using a single-item question, commonly used in large population samples. Finally, study (Özteke Kozan and Kesici, 2023) did not use a standardised questionnaire; instead, it applied a qualitative phenomenology approach to investigate participants’ experiences of death anxiety during the COVID-19 pandemic.

## Discussion

### Main findings and interpretive overview

The scarcity of studies identified throughout this review may reflect not only the methodological inclusion criteria but, more importantly, the still inconsistent scientific exploration of the topic of mortality awareness in treatment adherence in older adults with chronic diseases. These findings highlight a significant research gap in the literature examining this topic and underscore the importance of addressing this phenomenon more systematically.

The purpose of this integrative review was to examine how death anxiety influences psychological and behavioural responses to chronic illness in older adults. The reviewed studies reveal a contradictory pattern: while some associate death anxiety with psychological distress and avoidance behaviours (Bharti and Bharti, 2024; Doğan and Hacıköylü, 2025; Comertoglu et al., 2024), others link it to proactive engagement and spiritual growth (Daaleman and Dobbs, 2010; Chopik, 2017; Ji et al., 2024). This ambivalence suggests that death anxiety functions more as a contextual amplifier than a direct determinant, with outcomes shaped by individual coping resources and sociocultural moderators (Rezaei Aderyani et al., 2021; Guo et al., 2024; Özteke Kozan and Kesici, 2023). These heterogeneous responses are consistent with theoretical models such as Terror Management Theory and Socioemotional Selectivity Theory (Hamidi et al., 2024; Pandya and Kathuria, 2021), which highlight how mortality awareness interacts with perceived control, time perspective, and existential meaning.

### Psychological and behavioural responses to mortality awareness

Although treatment adherence was discussed as a central issue in the Introduction, the results of this review provide further insight into its association with death anxiety. The psychological and behavioural responses identified (i.e., avoidance, denial, or proactive engagement) have direct relevance to adherence behaviours. Death anxiety appears to exert a dual influence on treatment adherence: in contexts of low emotional resilience or limited social support, it tends to provoke avoidance and disengagement (Bharti and Bharti, 2024; Rezaei Aderyani et al., 2021; Guo et al., 2024), conversely, when mediated by spirituality, meaning-making, or self-efficacy, it may foster proactive engagement with treatment (Daaleman and Dobbs, 2010; Ji et al., 2024). This ambivalence reinforces the need for integrative clinical models that incorporate existential dimensions into chronic illness management”.

Mortality awareness elicits diverse psychological and behavioural responses in older adults with chronic illnesses, shaped by factors such as resilience, social support, spirituality, and illness severity. Psychologically, heightened mortality awareness is often linked to emotional distress—such as despair, anxiety, and depression—particularly among individuals with low religiosity, poor health, or limited support (Bharti and Bharti, 2024; Daaleman and Dobbs, 2010; Doğan and Hacıköylü, 2025; Comertoglu et al., 2024). Behaviourally, it may lead to either avoidance (e.g., missed appointments, denial of illness) or proactive engagement (e.g., adherence, re-evaluation of priorities), depending on the individual’s coping resources and existential orientation (Daaleman and Dobbs, 2010; Chopik, 2017; Rezaei Aderyani et al., 2021; Guo et al., 2024; Ji et al., 2024).

At the psychological level, several studies have found that heightened mortality awareness often leads to increased emotional distress. This includes feelings of despair, anxiety, and depression, as well as a decline in overall psychological well-being. Notably, individuals with low intrinsic religiosity, limited social support, or poor health assessments tend to report greater difficulty in processing existential concerns.

Behaviorally, awareness of mortality can lead to very different outcomes. Some people may respond to death anxiety by avoiding it, such as postponing or refusing treatment, missing medical appointments, or minimising the severity of their illness. Conversely, others might adopt a more proactive approach, sticking to treatment plans, re-evaluating their life priorities, or seeking spiritual support.

These findings indicate that the psychological and behavioural effects observed in the reviewed studies are associated with death anxiety, an emotional response that may stem from awareness of one’s mortality. Rather than acting as a direct determinant, death anxiety functions as a psychological trigger whose effects are mediated by coping mechanisms and contextual factors. This interpretation aligns with Terror Management Theory and Socioemotional Selectivity Theory, which suggest that mortality awareness influences behaviour through symbolic defences, perceived time horizons, and emotional regulation (Carstensen et al., 1999; Carstensen et al., 2003; Chen et al., 2025; Gyimes and Valentini, 2023; Carstensen, 2021; Iverach et al., 2014).

### The role of spirituality and meaning-making

Spirituality has consistently emerged, in the studies analysed, to be a significant protective factor against the psychological and behavioural effects of death anxiety. In religious groups, those who engaged more deeply with their spirituality reported feeling less anxious about death and enjoyed greater emotional well-being (Ji et al., 2024). Similarly, study (Daaleman and Dobbs, 2010) found that individuals with stronger beliefs about the meaning of life and death adapted better psychologically and were more likely to follow their treatment plans.

Beyond the emotional support, spirituality also seems to boost resilience and acceptance of life’s big questions. Study (Özteke Kozan and Kesici, 2023), which used a qualitative phenomenological approach, found that spiritual beliefs gave participants a sense of peace and control, enhancing their ability to cope with the challenges of chronic illness. Study (Rezaei Aderyani et al., 2021) also found that religiosity was a strong predictor of lower anxiety and greater acceptance of finitude. However, spirituality is not universally protective; in rigid or guilt-inducing religious contexts, it may intensify emotional ambivalence or existential distress, particularly when beliefs conflict with illness experiences.

These findings support the idea that finding meaning and having spiritual guidance can help mitigate the emotional and behavioural impacts of death awareness. In this context, spirituality acts not only as a belief system but as a valuable resource that helps individuals integrate mortality into a meaningful life story, aiding emotional regulation and encouraging proactive health behaviours. This aligns with existential theories that emphasise the importance of meaning, purpose, and transcendence in dealing with the reality of human finitude (Pyszczynski et al., 2021; Shakil et al., 2022; Gomes, 2008; da Silva et al., 2024; Yalom, 2008).

### Sociodemographic patterns in death anxiety

The studies reviewed suggest that death anxiety in older adults dealing with chronic illnesses is strongly shaped by sociodemographic factors, especially gender, education, health status, and social vulnerability. Interestingly, women were consistently found to report higher levels of death anxiety compared to men (Bharti and Bharti, 2024; Guo et al., 2024; Ji et al., 2024). This gender disparity is corroborated by previous research, which often finds that women are more emotionally expressive and more likely to reflect on existential issues (Russac et al., 2007; Adelirad et al., 2021).

The higher levels of death anxiety observed in women may reflect greater awareness of health risks and a stronger inclination toward preventive behaviours. Prior studies have shown that older women tend to use health services more frequently and follow treatment recommendations better than men (Thompson et al., 2016; Cameron et al., 2010). Thus, in some cases, anxiety about death can serve not only as a sign of vulnerability, but also as a powerful motivator to remain vigilant about health and take care of oneself (Dutta and Kaur, 2015; Goldenberg and Arndt, 2008).

Lower educational attainment, poor self-rated health, and financial hardship were consistently associated with higher death anxiety (Daaleman and Dobbs, 2010; Doğan and Hacıköylü, 2025; Comertoglu et al., 2024). These factors contribute to social frailty and reduced access to health resources, which, when combined with limited coping strategies, may lead to treatment abandonment or maladaptive behaviours (Goldenberg and Arndt, 2008; Bunt et al., 2017; Ebrahimi et al., 2018).

In addition, living in rural areas and having limited access to healthcare were identified as factors that increase vulnerability to death-related concerns (Rezaei Aderyani et al., 2021; Comertoglu et al., 2024). Older adults in these regions often face geographical isolation, a lack of specialised care, and minimal psychosocial support. All of these factors can amplify existential fears and make it more difficult to cope with chronic illness (Goins et al., 2005; Song et al., 2022). These findings suggest that death anxiety is not just a personal or emotional issue, but it is influenced by broader social and structural factors that affect how people age and cope with illness. Health disparities related to education, income, and access to healthcare—especially in rural or disadvantaged areas—significantly shape the psychological experience of ageing (Reilly, 2021; World Health Organization, 2008). Death anxiety is not merely a personal emotional response, but a phenomenon deeply embedded in structural inequalities that shape the ageing experience. Addressing these disparities is essential for promoting equitable and psychologically informed care for older adults.

### Assessment of death anxiety

Although anxiety about death was a central theme in the studies analysed, the way it was measured varied considerably. Four studies (Bharti and Bharti, 2024; Rezaei Aderyani et al., 2021; Guo et al., 2024; Comertoglu et al., 2024) were based on Templer’s Death Anxiety Scale (DAS), while two others (Doğan and Hacıköylü, 2025; Ji et al., 2024) used culturally adapted versions (TDAS, CT-DAS). Although the DAS is frequently used, it has been criticised for being one-dimensional and not fully capturing the existential and cultural complexities experienced by older adults (Dadfar et al., 2021; Sharif Nia et al., 2021). This limitation is particularly relevant in older populations, where cultural, existential, and emotional nuances require more sensitive and multidimensional assessment tools.

On the other hand, two studies (Daaleman and Dobbs, 2010; Ji et al., 2024) adopted a more comprehensive approach, using multidimensional tools—the Death Attitude Profile-Revised and the Collett-Lester Fear of Death Scale. These tools provided a deeper insight into feelings of fear, acceptance, and avoidance. One study (Chopik, 2017) opted for a single-item measure, which is quick and straightforward but does not delve deeply into the concepts. In contrast, a qualitative study (Özteke Kozan and Kesici, 2023) offered rich contextual insights without relying on standardised measures. The diversity of instruments used across studies complicates cross-study comparisons and may influence the identification of clinically relevant patterns, especially in culturally diverse settings.

In summary, the studies we reviewed explored various facets of the death-related experience — such as fear, denial, meaning-making, and emotional regulation — often without clear definitions. Only a few made a point of differentiating death anxiety from similar concepts, such as mortality awareness or existential anxiety (Menzies et al., 2022; Abdel-Khalek, 1998). These inconsistencies highlight the urgent need for internationally validated, multidimensional instruments that can reliably differentiate between death anxiety, mortality awareness, and existential distress in older adults.

### Theoretical and clinical implications

This review highlights how anxiety about death plays a crucial role in shaping the emotional and behavioural responses of older adults coping with chronic illness. Rather than having a singular effect, anxiety about death interacts with personal resources such as spirituality, self-efficacy, and the ability to find meaning, as well as with the broader social context and sociodemographic factors. Theoretical models such as Terror Management Theory and Socioemotional Selectivity Theory offer valuable insights into how mortality awareness influences health behaviours. TMT explains defensive reactions to death anxiety, while SST highlights how perceived time horizons shift motivational priorities in later life—both with direct implications for clinical strategies. These ideas point to the importance of developing theoretical models that consider how this emotional response can block or promote engagement with treatment (Iverach et al., 2014; Tomer and Eliason, 1996; Horgan et al., 2024).

Acceptance and Commitment Therapy (ACT) offers a valuable framework for this. By promoting psychological flexibility—the ability to accept distressing internal experiences while remaining true to personal values—ACT can help older adults respond more effectively to the suffering that accompanies thoughts about death (Hayes, 2004). From this perspective, death anxiety is not just something to be eliminated; it is a signal that can guide individuals toward meaningful, value-driven actions (Nejad-Ebrahim Soumee et al., 2024; Bayati et al., 2017). In addition to ACT, brief existential interventions such as meaning-centered therapy and dignity therapy have shown promise in addressing death anxiety and enhancing psychological well-being in older adults with chronic illness.

This perspective encourages the creation of integrative models that connect death anxiety to health behaviours, helping to clarify when and how this emotional response can lead to avoidance or active engagement. Future theoretical work will further explore this conceptual direction. These models may help clinicians identify when death anxiety is likely to hinder adherence and when it can be harnessed to motivate engagement with treatment.

### Recommendations for future research

Future research should focus on creating and validating robust, multidimensional tools that can effectively differentiate between cognitive, emotional, and behavioural reactions to death anxiety, especially in the context of ageing and chronic illness (Hernandez et al., 2024).

In addition, it is crucial to culturally diversify research samples. Many studies included in this review were limited to specific geographical areas, with no representation from places such as continental Europe, Africa, and indigenous communities. Understanding how cultural values and belief systems influence the experience of death anxiety and its effects on treatment adherence is a significant gap that needs to be addressed (Gysels et al., 2020).

Furthermore, future studies should investigate the effectiveness of psychological interventions—particularly those that combine Social Emotional Selectivity Theory (SST) with clinical approaches such as Acceptance and Commitment Therapy—in alleviating death-related distress and encouraging value-driven engagement in health behaviours.

### Limitations

This review highlights several strengths, such as the combination of existential and behavioural perspectives in a cohesive theoretical framework. It also presents a clear and systematic approach to identifying and evaluating studies, offers broad coverage of databases without time limits, and presents a synthesised evidence that can be useful in both clinical and public health contexts. However, there are some limitations worth mentioning. The limited cultural diversity of the included studies—most conducted in Turkey, China, and the US—may constrain the applicability of findings to other sociocultural contexts. This gap highlights the importance of expanding research to underrepresented regions and belief systems (Gysels et al., 2020).

Second, there was significant variation in the methods and tools used to measure death anxiety, spirituality, and related concepts. This inconsistency makes it difficult to compare results across studies and complicates the overall synthesis of conclusions (Menzies et al., 2022; Galvao and Pereira, 2014). Thirdly, the specific nature of the study samples also affects generalisation. Moreover, the predominance of cross-sectional designs limits the ability to understand how death anxiety and coping mechanisms evolve throughout the ageing process. Many studies focused on specific subgroups — such as older adults with cardiovascular problems or those living in rural areas — leaving out the broader diversity of older adults dealing with chronic diseases in various socioeconomic contexts. Future research should also prioritise longitudinal designs to capture the evolution of death anxiety over time, and mixed-methods approaches to integrate quantitative patterns with rich qualitative insights. Intervention studies testing existential and ACT-based therapies could further clarify their impact on adherence and psychological well-being.

Fourth, although the database search was exhaustive, some relevant studies may have been overlooked if they were not indexed in the chosen databases or published in languages other than English, Portuguese, or Spanish.

Finally, most studies relied on self-assessment questionnaires, which may be influenced by biases such as social desirability and underreporting — especially on sensitive topics such as death (Paulhus, 2017). Future research could greatly benefit from using a combination of methods, combining self-assessments with qualitative interviews, implicit or physiological measures, or observational techniques (Paulhus, 2017; Singh et al., 2022). Although these limitations restrict the generalisability of the findings, they underscore the urgent need for methodologically robust, culturally diverse, and longitudinal research to deepen our understanding of death anxiety in ageing.

## Conclusion

This integrative review highlights how death anxiety plays a significant role in shaping the psychological and behavioural responses of older adults coping with chronic illness. Rather than being a single emotional reaction, death anxiety is actually a complex phenomenon influenced by various factors, such as psychosocial resources, personal experiences, and how individuals perceive their remaining time.

The results reveal that death anxiety can lead to different outcomes: it can trigger avoidance behaviours, such as denial and abandonment of treatment, or it can encourage positive actions, such as proactive self-care and acceptance of existential issues. These outcomes appear to be influenced by factors such as spirituality, self-esteem, social support, and the burden that the illness represents for the individual.

Overall, this review emphasises the importance of developing theoretical and clinical strategies that consider the complex relationship between emotions related to death and health behaviours in older adults. These findings reinforce the need for clinical interventions that address the existential dimension of treatment adherence. By recognising death anxiety as a central psychological dimension of chronic illness management, healthcare systems can move beyond purely biomedical approaches and integrate interventions that enhance both adherence and existential well-being in older adults.

## Appendix 1

### Data extraction form

**Table:**

Field	Description
Study title	Title of the included study.
Authors	Names of the authors of the study.
Country	Country where the study was conducted.
Year	Year of publication.
Study type	Type of study (e.g., qualitative, quantitative, mixed-methods).
Level of evidence	Classification of the study's evidence level.
Objective	Main objective of the study.
Chronic disease	Type of chronic illness addressed.
Participants	Characteristics of the study population.
Concept Related to finitude	Concepts such as death anxiety, mortality awareness, existential distress.
Variables analyzed	Main variables studied.
Theoretical perspective	Theoretical framework used in the study.
Main findings	Summary of the key results.
Limitations	Limitations reported by the authors.
