# Supplementary material for: Psychological and behavioural responses to death anxiety in older adults with chronic illnesses: a systematic integrative review
Source: Front Psychol. 2025 Dec 10;16:1684385. doi: 10.3389/fpsyg.2025.1684385 (PMC12728577; doi:10.3389/fpsyg.2025.1684385)
Supplement: Supplementary file 1 [file Table_1.docx]

**Supplementary Table 1.** Bibliographic database search strategies.

| **Database** | **Search terms** | **Results (number of articles, n)** |
| --- | --- | --- |
| PubMed | ("Death Attitudes"[Title/Abstract] OR "Mortality Salience"[Title/Abstract] OR "Death Anxiety"[Title/Abstract] OR "Death Awareness"[Title/Abstract] OR Finitude[Title/Abstract]) AND ("Chronic Illness"[Title/Abstract] OR "Chronic Disease"[Title/Abstract] OR "Chronic Condition"[Title/Abstract]) AND (elder*[Title/Abstract] OR "older adults"[Title/Abstract] OR "aging"[Title/Abstract]) | n=8 |
| Web of Science | TS=("Death Attitudes" OR "Mortality Salience" OR "Death Anxiety" OR "Death Awareness" OR Finitude) AND TS=("Chronic Illness" OR "Chronic Disease" OR "Chronic Condition") AND TS=(elder* OR "older adults" OR "aging") | n=15 |
| Scopus | TITLE-ABS-KEY ( "Death Attitudes" OR "Mortality Salience" OR "Death Anxiety" OR "Death Awareness" OR finitude ) AND TITLE-ABS-KEY ( "Chronic Illness" OR "Chronic Disease" OR "Chronic Condition" ) AND TITLE-ABS-KEY ( elder* OR "older adults" OR "aging" ) | n= 38 |
| PsycINFO | (TI,AB("Death Attitudes") OR TI,AB("Mortality Salience") OR TI,AB("Death Anxiety") OR TI,AB("Death Awareness") OR TI,AB(Finitude)) AND (TI,AB("Chronic Illness") OR TI,AB("Chronic Disease") OR TI,AB("Chronic Condition")) AND (TI,AB(elder*) OR TI,AB("older adults") OR TI,AB("aging")) | n= 5 |
| Total | | n=66 |
| Total after removing duplicates | | n=45 |

|  |
| --- |
