# Supplementary material for: Psychological and behavioural responses to death anxiety in older adults with chronic illnesses: a systematic integrative review
Source: Front Psychol. 2025 Dec 10;16:1684385. doi: 10.3389/fpsyg.2025.1684385 (PMC12728577; doi:10.3389/fpsyg.2025.1684385)
Supplement: Supplementary file 2 [file Table_2.docx]

| **Study Number** | **Author** | **Type of study** | **Level of evidence** |
| --- | --- | --- | --- |
| 36 | Akanksha Bharti and Das Ambika Bharti (2024) India | Cross-sectional Study | 4/8 = 50% Medium |
| 37 | Timothy P. Daaleman and Debra Dobbs (2010) USA | Cross-sectional Study | 8/8 = 100% High |
| 38 | William J. Chopik (2017) USA | 1. Cross-sectional Study/ 2. Longitudinal | 8/8 = 100% High  8/11 = 88% High |
| 39 | Rezaei Aderyani, Mohsen, et al. (2021) Iran | Cross-sectional Study | 8/8 = 100% High |
| 40 | Nurhan Dogan and Kerime Hacıkoylü (2025) Turkey | Cross-sectional and correlational study | 8/8 = 100% High |
| 41 | Guo, Jin, et al. (2024) China | Longitudinal Survey | 7/11 = 77% High |
| 42 | Özteke Kozan, H. İ., & Kesici, Ş. (2023). Turkey | Phenomenological qualitative research | 8/10 = 80% High |
| 43 | Ji, Pengjuan et al. (2024) China | Cross-sectional Study | 8/8 = 100% High |
| 44 | Odaci Comertoglu, Ezgi et al. (2024) Turkey | Cross-sectional Study | 8/8 = 100% High |

**Supplementary Table 2:** Quality assessment according to the Joanna Briggs Institute Critical Appraisal Tools
